# Diseases of Rectum and Anus

**Published:** 1896-01

**Authors:** 


DISEASES OF RECTUM AND ANUS. By Gant and Allingham. Published by The F. A. Davis Co., Philadelphia.

By S. G. Gant, M. D., Professor of Rectal and Anal Surgery in the University Medical College, Kansas City; Lecturer on Rectal and Anal Diseases in the Scarritt Training School and Hospital for Nurses, etc.; and H. W. Allingham, M. D., Surgeon to the Great Northern Hospital, and Junior Surgeon to St. Mark's Hospital for Rectal Diseases, London, etc. With numerous illustrations, including several full-page colored photo-engravings. Royal Octavo. In press. Ready in February.

